# The Histone Acetyltransferase MOF Regulates SIRT1 Expression to Suppress Renal Cell Carcinoma Progression

**DOI:** 10.3389/fonc.2022.842967

**Published:** 2022-02-16

**Authors:** Renbo Guo, Yiran Liang, Benkui Zou, Danyang Li, Zhen Wu, Fei Xie, Xu Zhang, Xiangzhi Li

**Affiliations:** ^1^ Shandong Provincial Key Laboratory of Animal Cell and Developmental Biology, School of Life Sciences, Shandong University, Qingdao, China; ^2^ Department of Urology, Shandong Cancer Hospital and Institute, Shandong First Medical University and Shandong Academy of Medical Sciences, Jinan, China; ^3^ Department of Breast Surgery, General Surgery, Qilu Hospital of Shandong University, Jinan, China; ^4^ Rehabilitation Center, Qilu Hospital, Cheelo College of Medicine, Shandong University, Jinan, China

**Keywords:** renal cell carcinoma, MOF, SIRT1, progression, tumor suppressor

## Abstract

**Background:**

Renal cell carcinoma (RCC) is one of the most common and lethal human urological malignancies around the world. Although many advancements in diagnostic and therapeutic strategies have been acquired, the prognosis of patients with metastatic RCC was poor. Thus, there is an urgent need to understand the molecular mechanism of RCC.

**Methods:**

The quantitative real-time PCR (qRT-PCR) was used to detect the RNA expression of MOF in human RCC tissues and cell lines. The protein expression of MOF was analyzed with immunohistochemistry (IHC) and Western blot. To understand the regulatory mechanism of MOF in liver cancer, ChIP-qPCR assay and dual-luciferase assay were performed. Moreover, a series of *in vivo* and *in vitro* experiments were conducted to evaluate the effect of MOF on renal cell carcinoma progression.

**Results:**

In the present study, we found that Males absent on the first (MOF), a histone acetyltransferase involved in transcription activation, was significantly decreased in both RCC tissues and RCC cells compared to normal tissues and non-cancer cells. Moreover, MOF downregulation was associated with advanced histological grade, pathologic stage and distant metastasis of RCC patients. Ectopic expression of MOF could significantly attenuate cell proliferation and promote cell apoptosis. Besides, MOF overexpression also suppressed migration of RCC cells through inhibiting epithelial-mesenchymal transition (EMT). Importantly, the inhibition of tumor growth by MOF was further confirmed by *in vivo* studies. Mechanism dissection revealed that MOF could transcriptionally upregulate the expression of SIRT1, leading to attenuated STAT3 signaling, which was involved in cell proliferation and migration. Moreover, SIRT1 knockdown could restore the biological function induced by MOF overexpression.

**Conclusions:**

Our findings indicated that MOF serves as a tumor suppressor *via* regulation of SIRT1 in the development and progression of RCC, and MOF might be a potent biomarker for diagnosis and prognosis prediction of RCC patients.

## Introduction

Renal cell carcinoma (RCC) is a malignant tumor arising from urinary tubular epithelial cells (1), accounting for more than 90% of tumors in human kidney. It was reported that there were 403,262 new cases and 175,098 deaths in 2018 (2) and the incidence of RCC is gradually increasing every year. Radical nephrectomy is the major effective treatment for patients with RCC due to the poor effect to chemotherapy and radiotherapy (3). Moreover, although significant advances have been acquired in therapeutic strategies, including modified surgical techniques and improved systemic treatment with targeted agents, the prognosis of RCC is still far from satisfactory because of the tumor recurrence and metastasis (4). The median survival time of patients with metastatic RCC was only 13 months (5). Therefore, comprehension of the molecular mechanisms of RCC carcinogenesis and progression is essential for detecting diagnostic and therapeutic biomarkers.

Histone acetylation modification is one of the most significant epigenetic modifications involved in various cellular biological process (6), such as genetic transcription, chromosome constitution, cell cycle control, and DNA damage repair. The balance of global histone acetylation modification is regulated by histone acetyltransferases (HATs) and histone deacetylases (HDACs) (7), and its disturbance shows close association with the initiation and progression of various cancers. For example, the total acetylation levels of histone H3 were negatively correlated with Fuhrman grading, pT-stage, and distant metastasis of RCC (8), whereas the alteration of global H4K16ac was closely associated with the occurrence of tumors (9), indicating the diagnostic and prognostic value.

MOF, as a member of the MYST family of HATs in human cells, is responsible for H4K16ac (10). Depletion of MOF not only leads to global reduction of H4K16ac, but also influences various physiological and pathological processes (11–13), including cell proliferation, DNA damage repair, chromatic constitution, gene transcription, stem cell self-renewal, and embryonic development. Recently, mounting evidences revealed that abnormal expression of MOF was involved in various primary cancers, however, the expression patterns of MOF were varied among different cancers. The expression of MOF was upregulated in lung non-small cell lung cancer (NSCLC) tissues compared to normal tissues, and MOF overexpression led to enhanced cell proliferation, migration, adhesion, and drug resistance of NSCLC cells (14, 15). On the contrary, MOF was reported to be downregulated in multiple cancers, including breast cancer (16), medulloblastoma (17), gastric cancer (18), and ovarian cancer (19). Although the downregulation of MOF had been found in RCC (18, 20), it remains poorly understood about the functions and molecular mechanisms of MOF in RCC.

In this study, we assess the expression of MOF in RCC tissues and RCC cells, and further evaluate the association between MOF expression and corresponding clinicopathological features. In addition, the role of MOF in regulating RCC cell proliferation and mobility and the underlying mechanism was also investigated. Our results would help to comprehensively understand the function of MOF in RCC and provide a novel biomarker for diagnosis and treatment in RCC patients.

## Material and Methods

### Human Samples

A total of 52 RCC patients undergoing surgery in the department of urology at the Shandong Cancer Hospital and Institute were included in the present study. The RCC tissues and paired adjacent normal tissues were stored at − 80°C until use. None of the patients received chemotherapy or radiotherapy before surgery. Written informed consent was obtained from all participants, and all the experimental procedures were approved by the Ethics Committee of Shandong Cancer Hospital and Institute.

### Gene Expression Profiles

The gene expression data matrix of normal tissue and RCC tissue was obtained from the GEO database (https://www.ncbi.nlm.nih.gov/geo/), which is accessible through the GEO platforms GPL570 (GSE53757).

### Cell Culture

The human RCC cell lines, 786-O, Caki-1, 769-P, A498, ACHN and the immortalized proximal tubule epithelial cell line from normal adult human kidney (HK-2) were purchased from American Type Culture Collection (ATCC, Manassas, VA, USA). Mycoplasma detection was performed using a Mycoplasma Detection Set (Takara, Shiga, Japan) for all the cells. All the RCC cell lines were routinely cultured in Dulbecco’s modified Eagle’s medium (DMEM, Invitrogen, Carlsbad, CA, USA) supplemented with 10% fetal bovine serum (FBS), 100 U/ml Penicillin, and 100 µg/ml streptomycin in the humidified atmosphere with 5% CO2 at 37°C.

### Cell Transfection

To overexpress MOF, pcDNA3.1-MOF vector containing the open reading frame (ORF) of MOF was used. Empty vector was used as a control. G418 (2mg/ml) was used for generating stably transfected cells. To knockdown MOF in RCC cells, duplexes of siRNA targeting MOF and negative control synthesized by GenePharma (Shanghai, China) were used. The cells were transfected using the Lipofectamine 2000 (Invitrogen) according to the manufacturer’s instructions.

### RNA Extraction and qRT-PCR

Total RNAs were isolated from tissues or cells using Trizol reagent (Invitrogen, Grand Island, USA) according to the manufacturer’s instructions. One microgram of total RNA was subjected to reverse transcription using PrimeScript reverse transcriptase (RT) reagent kit (Takara, Shiga, Japan). Then, quantitative reverse transcriptase PCR (qRT-PCR) was conducted using SYBR green (Takara, Shiga, Japan) to determine the RNA expression level. Actin was used as the internal control, and expression of RNA was calculated by the relative quantification using 2^−ΔΔCT^ method.

### Western Blotting (WB)

Total proteins of tissues or cells were extracted by RIPA lysis buffer (Beyotime, China) containing protease inhibitor cocktail (MedChemExpress, China) according to the manufacturer’s instructions. Then proteins were subjected to SDS-PAGE and then transferred to 0.22 μm PVDF membranes (Millipore, MA, USA). After being blocked with 5% skim milk powder and incubated with primary antibodies: anti-GAPDH (PTG, 60004-1-Ig), anti-MOF (Abcam, ab72056), anti-Fibronectin (PTG, 15613-1-AP), anti-N-cadherin (PTG, 22018-1-AP), anti-E-cadherin (PTG, 20874-1-AP), anti-Vimentin (PTG, 60330-1-Ig), anti-SIRT1 (Abcam, ab110304), anti-STAT3 (CST, 9139). Then, the membranes were incubated with HRP-conjugated anti-mouse (CST, 7076) or anti-rabbit (CST, 7074) secondary antibodies and detected through ECL detection system (Bio-Rad, USA). GAPDH was used as the internal control.

### Cell Proliferation Assay

Transfected RCC cells were seeded in 96-well plates (1500 cells/well) and cultured for indicated time. 20 μl of 5 mg/ml MTT was added into each well and incubated for another 4 h in incubator at 37°C. After removal of the media, 100 μl DMSO was added. Then the absorbance was measured at 570 nm on a Microplate Reader (Bio-Rad) and the proliferation curves were calculated.

### Colony Formation Assay

Transfected RCC cells were seeded in 6 cm plate (500 cells/plate) and cultured for 10-14 days. Then cell colonies were washed by PBS, fixed with ethanol and stained by crystal violet. The colonies were taken pictures and counted.

### EdU Incorporation Assay

Transfected RCC cells were seeded in 96-well plates (1×10^4^ cells/well) and incubated with 50 μM EdU for 2.5 h. The EdU Proliferation Kit (RiboBio Guangzhou, China) was used to evaluate cell proliferation viability following the standard protocol. Images were taken using an Olympus microscope (Olympus, Tokyo, Japan).

### Cell Migration Assays

The invasive capability of RCC cells was determined by the transwell migration assays. 5 × 104 of infected RCC cells were harvested and seeded with serum-free DMEM into the upper chambers, and the bottom chambers were filled with medium containing 20% FBS. After incubation for 24 h at 37°C, the cells attached to the lower surface of the membrane were fixed by 4% methanol and stained with 0.1% crystal violet. Cells were counted and photographed using an Olympus light microscope.

### Wound Healing Assay

Indicated cells were plated to confluence in 24-well plates. Then streaks across the plate were made in the monolayer with a pipette tip. Images were captured at 0 and 24 h after wounding using an Olympus light microscope.

### Chromatin Immunoprecipitation (ChIP) Assay

ChIP assay was performed using the ChIP kit (CST, USA) according to the manufacturer’s instruction. Briefly, transfected RCC cells were crosslinked with 4% formaldehyde for 10 min followed by sonication to yield genomic DNA fragments with an average length of 200-1000 bp. The lysates were then immunoprecipitated with anti-MOF antibodies (Abcam, Cambridge, UK) or normal rabbit IgG. The immunoprecipitated DNA was detected by qRT-PCR and the enrichment was expressed as fold enrichment compared to IgG.

### Luciferase Assay

The SIRT1 gene promoter segment covering from -2000 bp to +1 bp was cloned into the pGL3-basic vector (Promega, Madison, WI, USA), which was termed as pGL3-SIRT1. The vector of pcDNA3.1-MOF was co-transfected with pGL3-SIRT1 and pRL-TK vector using Lipofectamine 2000 (Invitrogen). Luciferase activity was measured by dual-luciferase assay system (Promega) according to the manufacturer’s manual.

### Immunohistochemistry

Tissues were embedded in paraffin and cut into 4 µm sections. The sections were de-paraffinized in xylene and rehydrated in a series of alcohol. Then antigen retrieval was performed in 10 mM sodium citrate buffer (pH 6.0) using microwave heating. Endogenous peroxidase was blocked with 3% hydrogen peroxide and 5% BSA was used to block nonspecific binding. The sections were incubated with the primary antibody for MOF (1:200, Abcam, ab72056) and SIRT1 (1:200, Abcam, ab110304) overnight at 4°C. After incubation with corresponding secondary antibodies at 37°C for 1 h, the sections were stained with diaminobenzidine (DAB) and counterstained with hematoxylin. The representative images were taken using an Olympus light microscope.

### Tumor Xenograft Formation and Lung Metastasis Model

Four-week-old nude mice were purchased from the Shanghai Experimental Animal Center and maintained in pathogen-free conditions. 769-P cells (1×10^7^ cells) stably transfected with pcDNA3.1-MOF or control vectors were subcutaneously injected into one flank of each mice (n=5). Tumor sizes were measured using digital calipers every five days, and tumor volumes were calculated using the formula: 1/2 × (length × width^2^). After 4 weeks, mice were killed, and tumors were excised and weighed. Hematoxylin and eosin (H&E) staining was performed for evaluation of tissue morphology and size of metastatic lesions. The animal experiments were conducted with approval from the Ethics Committee of Shandong Cancer Hospital and Institute.

### Statistical Analysis

Statistical analysis was performed using SPSS 21.0 (Chicago, IL, USA) and GraphPad Prism 8.0 software. Data were expressed as mean ± S.D. from three independent experiments. Student’s t-test was used for comparisons of two independent group and One-way ANOVA analysis was applied to compare statistical differences between groups. P-value < 0.05 was considered statistically significant.

## Results

### MOF Is Downregulated in RCC Tissues and Cells

**Figure 1:**
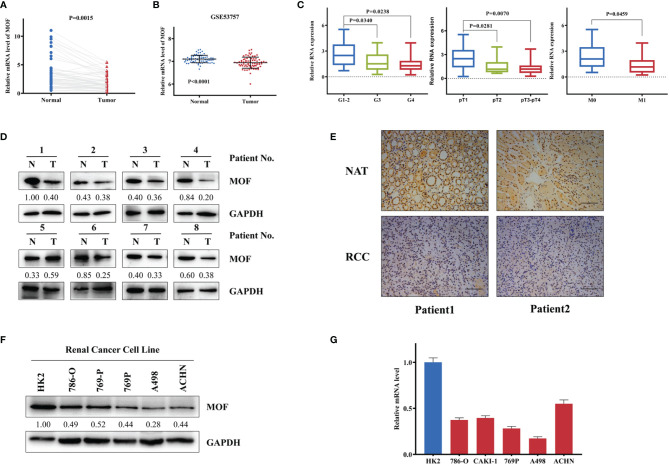
MOF is downregulated in renal cell carcinoma tissues and cells. **(A)** MOF RNA level is decreased in renal cell carcinoma tissues (n=52) compared to normal tissues (n=52). **(B)** MOF expression level is down-regulated in renal cell carcinoma tissues (n=72) compared to normal tissues (n=72) according to GSE53757 database. **(C)** The expression of MOF was decreased in renal cell carcinoma tissues with advanced histological grade, pathologic tumor stage and distant metastasis. **(D)** MOF protein level is decreased in most renal cell carcinoma tissues. **(E)** The expression of MOF is reduced in renal cell carcinoma tissues. Scale bar, 100 μm. **(F, G)** MOF protein **(F)** and RNA **(G)** level is down-regulated in renal cell carcinoma cells.

To investigate the role of MOF in RCC, the expression of MOF was analyzed in 52 paired RCC tissues and normal tissues. The qRT-PCR results revealed that the expression of MOF was significantly downregulated in RCC tissues compared to adjacent normal tissues (**Figure 1A**), which was consistent with the result (**Figure 1B**) obtained from GEO database (GSE53757). After analyzing the association between MOF expression and the clinicopathologic parameters, we found that low MOF expression level was significantly correlated with advanced histological grade, pathologic tumor stage and distant metastasis (**Figure 1C**). Moreover, the decreased expression of MOF in RCC tissues was further confirmed by western blot and immunohistochemistry (**Figures 1D, E**). Furthermore, we compared the levels of MOF in normal cells and RCC cells. Compared to normal renal tubular epithelial cells (HK2), the protein and mRNA levels of MOF in RCC cells (786-O, Caki-1, 769-P, A498, ACHN) were significantly decreased (**Figures 1F, G**). Taken together, MOF was significantly downregulated in RCC, and might serve as a tumor-suppressor in RCC.

### MOF Overexpression Inhibited the Proliferation of RCC Cells

**Figure 2:**
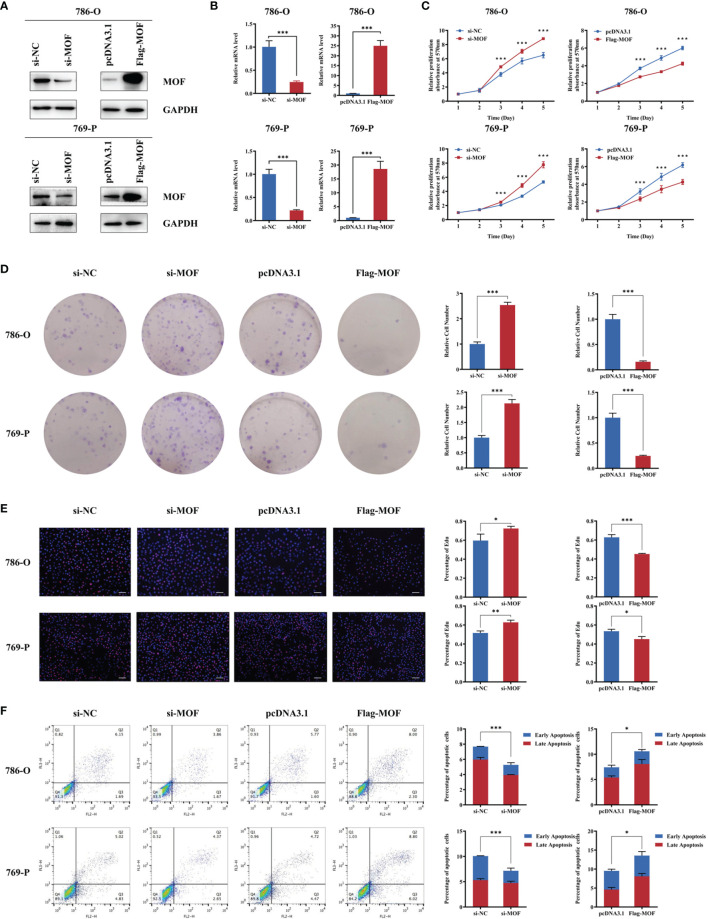
MOF overexpression inhibits renal cell carcinoma cell proliferation. **(A, B)** The knockdown or overexpression efficiency of MOF in protein **(A)** or RNA **(B)** levels in 786-O and 769-P cells. **(C)** MOF knockdown promoted renal cell carcinoma cell proliferation, and MOF overexpression inhibited renal cell carcinoma cell proliferation. **(D)** MOF knockdown led to increased renal cell carcinoma cell colony formation ability, whereas MOF overexpression caused inhibited renal cell carcinoma cell colony formation ability. **(E)** MOF knockdown promotes DNA replication of renal cell carcinoma cells, and MOF overexpression inhibited DNA replication of renal cell carcinoma cells. Scale bar, 100 μm. **(F)** MOF knockdown inhibited renal cell carcinoma cell apoptosis, and MOF overexpression promoted renal cell carcinoma cell apoptosis. (*, p < 0.05, **, p < 0.01, ***, p < 0.001).

We further investigated the potential functional role of MOF in RCC cells. The knockdown or overexpression efficiency of MOF in 786-O and 769-P cells were verified with western blot and qRT-PCR (**Figures 2A, B**). Both MTT assays and colony formation assays indicated that MOF knockdown led to increased proliferation of RCC cells, whereas MOF overexpression significantly inhibited the cell proliferation (**Figures 2C, D**). Moreover, EdU assays revealed that DNA synthesis activities of RCC cells were markedly increased after MOF knockdown and decreased after MOF overexpression (**Figure 2E**). Then flow cytometry analysis was performed to evaluate whether MOF could affect cell proliferation by modulating cell apoptosis. The results indicated that MOF knockdown led to a decreased apoptotic rate and ectopic expression of MOF promoted cell apoptosis (**Figure 2F**). These results suggested that MOF played an essential role in RCC cell proliferation.

### MOF Overexpression Depressed the Migration and Invasion of RCC Cells

**Figure 3:**
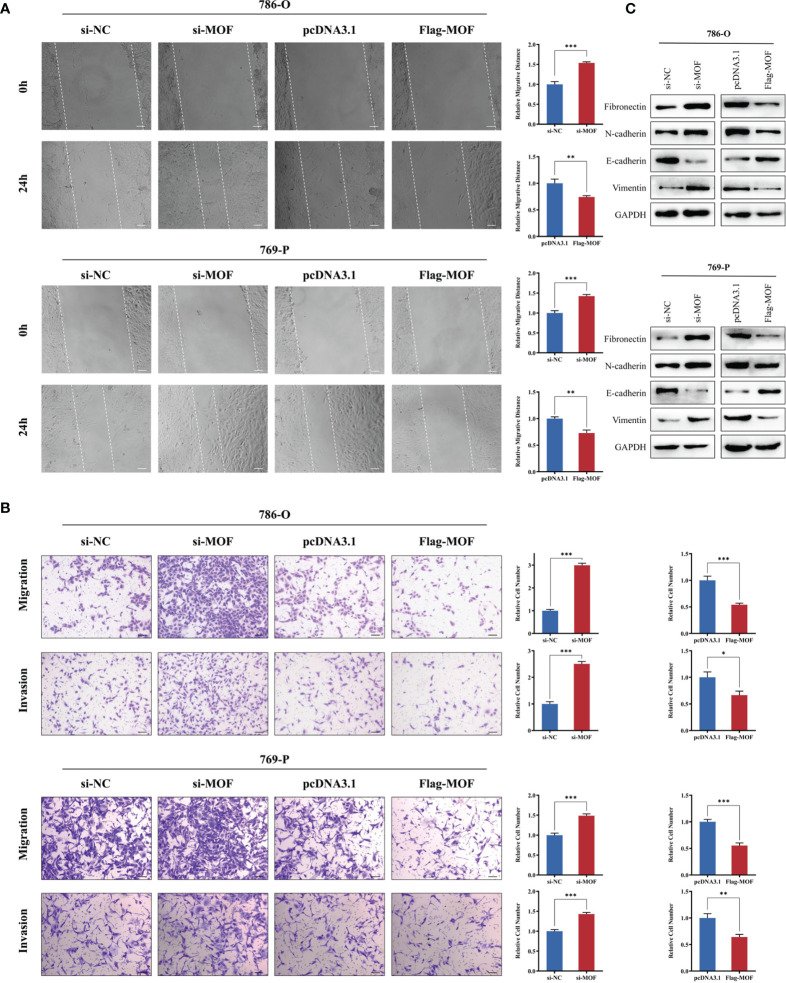
MOF overexpression inhibits renal cell carcinoma cell migration and invasion. **(A, B)** The wound healing assay **(A)** and transwell assay **(B)** indicated that MOF knockdown led to inhibited renal cell carcinoma cell migration, whereas MOF overexpression caused the opposite results. Scale bar, 100 μm. **(C)** Western blot was used to detect the effect of MOF on the expression of EMT markers. (*, p < 0.05, **, p < 0.01, ***, p < 0.001).

We then evaluated the effect of MOF on RCC cell motility. Knockdown of MOF observably increased the wound-healing ability of cells (**Figure 3A**). Consistently, the transwell migration and invasion assays revealed that MOF knockdown notably promoted the motility of RCC cells (**Figure 3B**). In accordance with the aforementioned results, MOF overexpression significantly attenuated cell migration and invasion (**Figures 3A, B**). Epithelial-mesenchymal transition (EMT) is one of the major mechanisms involved in cell malignant transformation, and our results indicated that MOF knockdown could decrease the expression of epithelial markers and increased the expression of mesenchymal markers, and MOF overexpression showed opposite effect on the expression of EMT-related proteins (**Figure 3C**). All these results indicated that MOF played an essential role in the migration and invasion of RCC cells.

### MOF Regulated SIRT1 and Its Downstream Genes in RCC Cells

**Figure 4:**
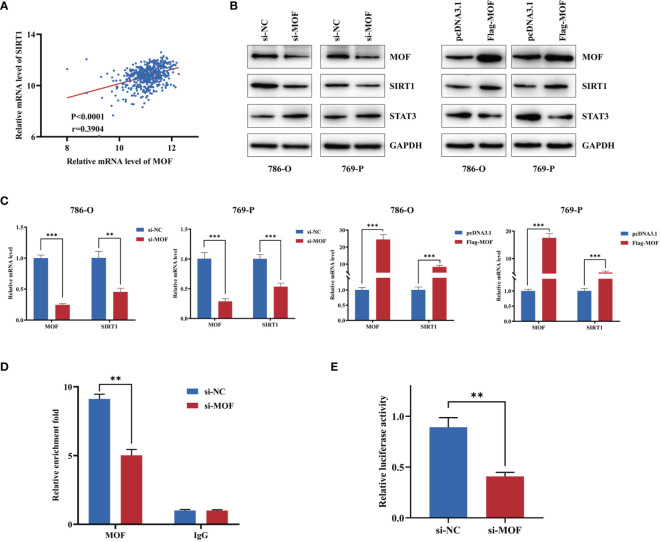
MOF regulates SIRT1 in renal cell carcinoma cells. **(A)** The expression of MOF was positively associated with the expression of SIRT1 based on TCGA database. **(B, C)** The effect of MOF on the protein **(B)** and RNA **(C)** expression levels of SIRT1 and its target genes were detected. **(D)** ChIP assay indicated that MOF could bind with the promoter of SIRT1. **(E)** Luciferase assay showed that MOF knockdown decreased the SIRT1 promoter activity. (**, p < 0.01, ***, p < 0.001).

SIRT1 was reported to play a crucial role in the development and progression of various cancers (21). We wondered whether MOF could regulate the expression of SIRT1 in RCC. Using TCGA starbase, we found a closely positive association between MOF and SIRT1 (**Figure 4A**). Moreover, our results indicated that MOF knockdown significantly reduced the protein and mRNA levels of SIRT1, leading to the upregulated expression of its downstream target gene, STAT3 (**Figures 4B, C**). Consistently, overexpression of MOF led to the opposite results (**Figures 4B, C**). To explore the role of MOF in regulating SIRT1 expression, ChIP assay was performed. The results showed that MOF could bind to the promoter of SIRT1 (**Figure 4D**). In addition, the luciferase report assay demonstrated that knockdown of MOF could attenuate the promoter activity of SIRT1 (**Figure 4E**). These results indicated that MOF could transcriptionally regulate the expression of SIRT1 in RCC cells.

### SIRT1 Contributed to the Biological Function of MOF in RCC Cells

**Figure 5:**
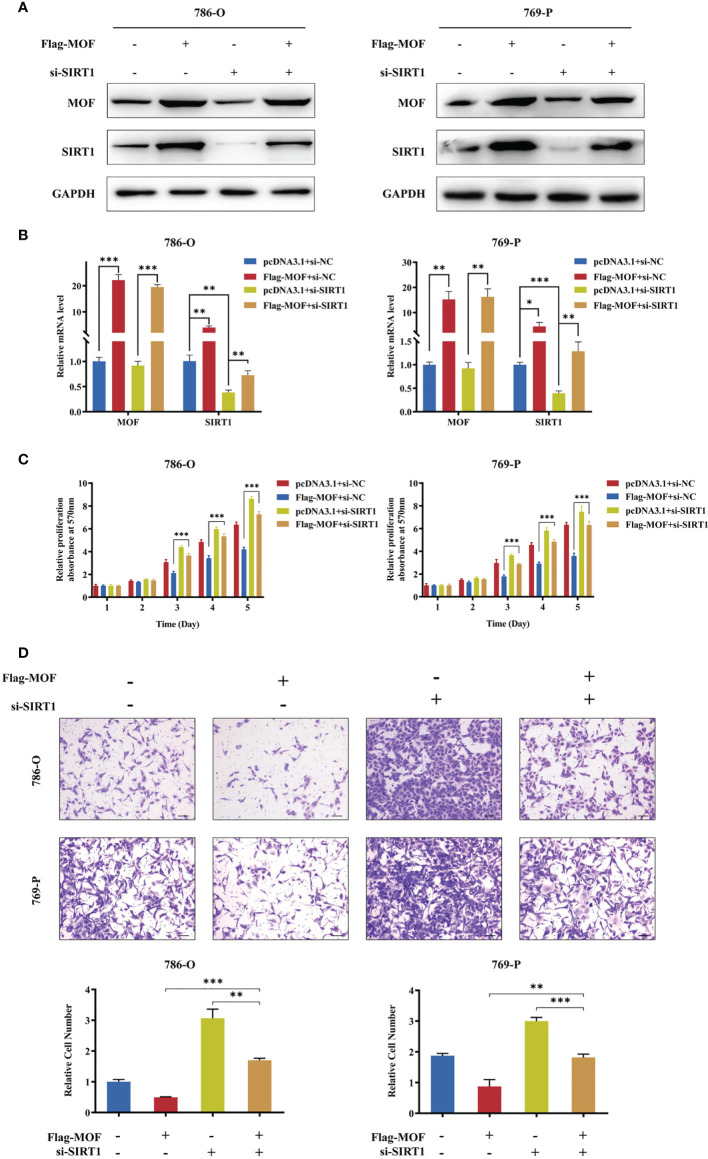
SIRT1 knockdown restored MOF-induced inhibition of cell proliferation and migration. **(A, B)** Western blot **(A)** and qRT-PCR **(B)** assays demonstrated the efficiency of SIRT1 knockdown and MOF overexpression in 786-O and 769-P cells. **(C)** SIRT1 knockdown restored the inhibited cell proliferation induced by MOF overexpression. **(D)** SIRT1 knockdown restored the inhibited cell migration induced by MOF overexpression. Scale bar, 100 μm. (*, p < 0.05, **, p < 0.01, ***, p < 0.001).

To further prove SIRT1 upregulation as a mediator of MOF in RCC cells, we performed rescue experiment by co-transfecting MOF overexpression vectors and siRNAs against SIRT1 into 786-O and 769-P cells. The transfection efficiency was confirmed by qRT-PCR and western blot (**Figures 5A, B**). The functional results showed that SIRT1 knockdown could rescued the attenuated cell proliferation and motility caused by MOF overexpression (**Figures 5C, D**). These data demonstrated that SIRT1 is a direct functional target of MOF in RCC.

### MOF Overexpression Attenuates RCC Growth and Progression *In Vivo*

**Figure 6:**
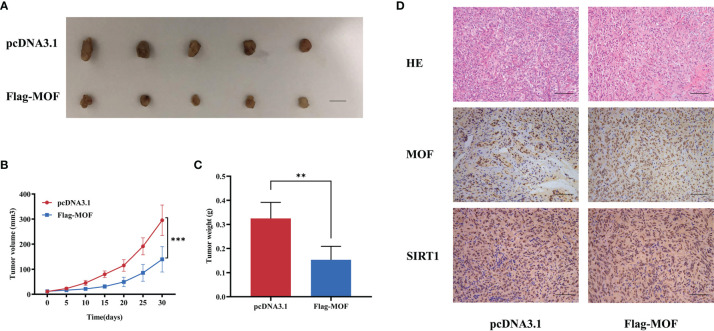
The effect of MOF on renal cell carcinoma cell growth *in vivo*. **(A)** Tumor xenograft model in nude mice. MOF-overexpressed 769-P cells and control cells were inoculated to the flank of nude mice. Scale bar, 1 cm. **(B, C)** The tumor volume **(B)** and tumor weight **(C)** were analyzed. **(D)** H&E staining showed the tissue morphology. Representative images of MOF and SIRT1 staining in the indicated tumor tissues. Scale bar, 100 μm. (**, p < 0.01, ***, p < 0.001.).

Furthermore, *in vivo* experiments were performed to evaluate the functions of MOF. The 769-P cells stably transfected with MOF overexpression vectors or control vectors were subcutaneously injected into nude mice, and the results showed that the growth rates and tumor weights were significantly decreased in MOF-overexpressed group compared to the control group (**Figures 6A–C**). Consistently, IHC analysis revealed the increased MOF and SIRT1 expression in the MOF-overexpressed group (**Figure 6D**). Our findings indicate that MOF overexpression in RCC cells inhibited tumor growth *in vivo*.

## Discussion

Even though extensive advancement in diagnosis and treatment of renal cell carcinoma have been made over the decades, metastasis and recurrence is still the intractable problem for affecting the patient prognosis. However, the detailed mechanism of metastasis is still poorly understood. Recently, increasing evidence suggested that the histone modification status in cells is significantly associated with the gene expression pattern, and abnormal global histone modification would further lead to cell dysfunction, even cancer. Various chromatin modifying enzymes, such as histone acetyltransferases (HATs) and histone deacetylases (HDACs) have been shown to participate in the tumorigenesis and progression of several cancers.

MOF (also called MYST1), a member of the MYST family of histone acetyltransferases (HATs), is the human ortholog of Drosophila male absent on the first (MOF) protein (22). The aberrant expression of MOF has been found in various cancers, such as breast cancer (16), ovarian cancer (19), and gastric cancer (18), functioning as an oncogene or tumor suppressor. However, the exact expression and role of MOF in renal cell carcinoma and the underlying mechanism were still unknown. The expression patterns of MOF in different cancers were varied. In this study, using a large number of renal cell carcinoma tissues from our center, we demonstrated that the RNA expression level of MOF was downregulated in renal cell carcinoma tissues, which is consistent with the previous report (20). Moreover, the expression analysis using GSE53757 database also indicated the decreased expression of MOF in renal cell carcinoma tissues. We further analyze the association between MOF expression and the clinicopathologic parameters, and found that downregulated expression of MOF was associated with advanced renal cell carcinoma, indicating the potential role of MOF in metastasis prediction. Consistently, the abnormal protein expression of MOF was also validated in renal cell carcinoma cells. These results indicated that MOF might play a tumor-suppressive role in renal cell carcinoma.

Previous studies show that MOF could regulate several cellular processes through modulating the status of histone H4K16ac (11, 23, 24), such as DNA damage repair, genomic instability, and gene transcription. However, the role of MOF in different cancers remains controversial. The expression of MOF was found to be higher in non-small cell lung cancer (NSCLC) tissues compared to corresponding normal tissues, and MOF overexpression led to enhanced proliferation, metastasis, and radiation resistance of NSCLC cells (14, 25). Another study reported that MOF was significantly upregulated at the protein level in hepatocellular carcinoma with microvascular invasion, and MOF downregulation would reduce the intravasation and metastasis *in vitro* and *in vivo* (26). On the contrary, the mRNA and protein levels of MOF were abnormally down-regulated in ovarian cancer tissues and cells, and the overexpression of MOF could inhibit the growth of ovarian cancer cells and promote cell apoptosis (27). Moreover, MOF-mediated H4K16Ac could promote the release of RNA polymerase II from pausing through recruiting BRD4 and pTEFb, and lead to reactivation of tumor suppressor TMS1 (16). These findings indicated that MOF had dual roles in cancers which largely depend upon the individual type of cancer itself with different cellular environment and various targets. In the study, we provided novel evidence for the tumor-suppressive role of MOF in renal cell carcinoma. The functional experiments demonstrated that MOF knockdown led to enhanced cell viability and motility, whereas overexpression of MOF inhibited cell proliferation and migration.

In order to discern the potential regulatory mechanism of MOF in renal cell carcinoma, we further explore its downstream pathway. Histone acetyltransferases (HATs) and histone deacetylases (HDACs) are major regulators for the overall level of acetylation in cells, which are in dynamic equilibrium and responsible for the function and expression of various genes (28). Aberrant expression of HATs and HDACs is associated with tumorigenesis and tumor development, and they are considered as novel anticancer targets for many cancers (29). Therefore, exploring the interaction between HATs and HDACs could help to find novel target for cancer treatment. The sirtuins (SIRT 1 to 7) belong to nicotinamide adenine dinucleotide (NAD+)-dependent class III HDACs with diverse roles in various biological activities (30, 31). Among them, SIRT1 is localized in cytoplasm and cell nucleus, and plays essential roles in the regulation of transcription factors and cellular metabolism through deacetylation of lysine residues (32, 33). Liu et al. revealed that MOF and SIRT1 were responsible for the acetylation level of WSTF, thus modulating the activities of WSTF and its effect on tumorigenesis (34). Moreover, SIRT1 could interact with MOF and deacetylate autoacetylated MOF, leading to increased recruitment of MOF to the chromatin and increased expression of its target gene HoxA9 (35). Another study reported that MOF could promote acetylation of DBC1 to inhibit DBC1-SIRT1 binding and increase the deacetylase activity of SIRT1, thus modulating cell response to DNA damage (36). These studies reveal that the interaction between MOF and SIRT1 serves as a significant mechanism in various biological processes and cancer development. However, the effect of MOF on SIRT1 expression and the functional role of SIRT1 in RCC remains unclear.

In this study, we found that the expression of MOF was positively associated with SIRT1 expression level. Knockdown of MOF led to decreased expression of SIRT1 in the protein and RNA levels, whereas MOF overexpression caused the opposite results. Further experiments indicated that, MOF could bind to the promoter of SIRT1, leading to the enhanced expression of SIRT1. Previous study reported that SIRT1 could deacetylate STAT3 and hence destabilize and negatively regulate STAT3 (21, 37). Consistently, our results also revealed that MOF overexpression led to decreased expression of the downstream target gene STAT3, whereas MOF knockdown upregulated the expression of STAT3. Multiple evidence found that the function of SIRT1 might be tissue or cell specific, and it could act as a tumor suppressor or oncogene in various cancers through regulating different biological pathways. Previous studies indicated that the expression of SIRT1 was upregulated in hepatocellular carcinoma tissues, and could directly deacetylate p62 to prevent its degradation (38). Moreover, SIRT1 expression was associated with tumor progression and poor prognosis in triple-negative breast cancer (39). In addition, SIRT1 overexpression led to enhanced expression of MMP2, promoting cell invasion in prostate cancer cells (40). On the contrary, the overexpression of SIRT1 in hormone receptor-positive patients and HER2+ patients were correlated with lower risks of lymph node metastasis (41). Moreover, the high expression level of SIRT1 was associated with a better survival rate in glioblastoma patients, and SIRT1 overexpression could enhance the inhibitory effect of Urolithin A on the tumor growth and metastasis of glioblastoma (42). Therefore, more efforts are needed to reveal the exact role of SIRT1 and its regulatory mechanism in RCC. In our study, the functional experiments indicated that knockdown of SIRT1 could promote the proliferation and migration of renal cancer cells, which further revealed that SIRT1 might act as an oncogene in renal cancer. Significantly, the inhibited effect of MOF overexpression on the proliferation and migration of renal cancer cells could be rescued by SIRT1 knockdown. These results further implicated that MOF might act as a tumor suppressor in renal cancer, partly through regulating the expression of SIRT1. Although our study and previous studies have revealed the significant role of MOF in tumor progression through modulating the expression of various genes (43, 44), such as SIRT6 and TNK2, more studies are needed to comprehensively understand the function and complex regulatory mechanism of MOF in the future.

In summary, our results indicated that MOF was downregulated in renal cell carcinoma tissues and cells, and the expression of MOF was negatively associated with the progression of renal cell carcinoma. We also identified the tumor-suppressive role of MOF *via* targeting SIRT1. These findings revealed that MOF might be a novel target for interventions in renal cell carcinoma.

## Conclusion

In the present study, we discovered the suppressive role of MOF in tumor progression through regulating the expression of SIRT1, and provides a potential therapeutic target for renal cell carcinoma.

## Data Availability Statement

The original contributions presented in the study are included in the article/supplementary material. Further inquiries can be directed to the corresponding author.

## Ethics Statement

The studies involving human participants were reviewed and approved by Ethics Committee of Shandong Cancer Hospital and Institute. The patients/participants provided their written informed consent to participate in this study. The animal study was reviewed and approved by Ethics Committee of Shandong Cancer Hospital and Institute.

## Author Contributions

RG and XL conceived and designed the experiments. RG, YL, DL, ZW, FX, and XZ performed the experiments. RG, YL, and BZ collected clinical samples. RG and YL analyzed the data. RG, YL, and XL wrote the manuscript. All authors read and approved the final manuscript.

## Funding

This work was supported by the National Key Research and Development Program of China (No. 2016YFE0129200), National Natural Science Foundation of China (No. 31571321), Natural Science Foundation of Shandong Province (No. ZR2019PH077 and ZR2020LZL011), Research Funds of the Shandong Provincial Key Laboratory of Animal Cell and Developmental Biology (No. SDKLACDB-2019010), and Key Scientific and Medical Project of Shandong (No. 2011QZ016 and 2016GSF201042).

## Conflict of Interest

The authors declare that the research was conducted in the absence of any commercial or financial relationships that could be construed as a potential conflict of interest.

## Publisher’s Note

All claims expressed in this article are solely those of the authors and do not necessarily represent those of their affiliated organizations, or those of the publisher, the editors and the reviewers. Any product that may be evaluated in this article, or claim that may be made by its manufacturer, is not guaranteed or endorsed by the publisher.
